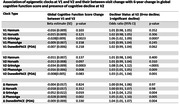# Longitudinal associations of epigenetic aging clocks with cognitive decline in diverse Hispanics/Latinos

**DOI:** 10.1002/alz.091514

**Published:** 2025-01-09

**Authors:** Rui Xia, Freddie Márquez, Wassim Tarraf, Linda C Gallo, Gregory A Talavera, Tamar Sofer, Charles Decarli, Hector M Gonzalez, Myriam Fornage

**Affiliations:** ^1^ University of Texas Health Science Center at Houston, Houston, TX USA; ^2^ University of California, San Diego, La Jolla, CA USA; ^3^ Wayne State University, Detroit, MI USA; ^4^ San Diego State University, San Diego, CA USA; ^5^ Harvard Medical School, Boston, MA USA; ^6^ University of California at Davis, Davis, CA USA; ^7^ University of California San Diego, San Diego, CA USA

## Abstract

**Background:**

Several epigenetic clocks based on DNA methylation (DNAm) have been developed to estimate an individual’s biological age. Age acceleration, the deviation of the DNAm‐estimated age from the chronological age, has been proposed as a novel biomarker to predict age‐associated conditions and life expectancy. Due to the paucity of longitudinal DNAm data, especially among diverse Hispanic/Latino adults, the association between changes in age acceleration over time and cognitive aging phenotypes has not been investigated.

**Method:**

We estimated epigenetic age acceleration from 5 PC‐based epigenetic clocks in 2667 Hispanic and Latino adults (58.2 years; 55.9% women) from the Hispanic Community Health Study/Study of Latinos (HCHS/SOL), who had available blood DNAm data and neurocognitive assessment at two visits (approximately 6 years apart). These included first generation clocks, Horvath and Hannum clocks; second generation PhenoAge and GrimAge; and third generation DunedinPACE (Pace of Aging). We used survey linear regression weighted least square models to estimate the association of each of these measures with change in general cognitive function score and cognitive decline status between the two visits. All models adjusted for age, gender, Hispanic background, and years of education.

**Result:**

For all epigenetic clocks, measures of age acceleration at visit 1 (V1) and visit 2 (V2) were strongly correlated (r=0.80 to 0.93; P <0.001). A higher GrimAge acceleration at V1 and V2 was associated with a greater decline in general cognitive function score between the two visits, while a higher V1 and V2 GrimAge acceleration and a higher V2 PhenoAge and DunedinPACE (POA) acceleration were associated with a greater likelihood of having cognitive decline at V2 (Table). An increase in GrimAge and PhenoAge acceleration between visits was associated with greater decline in global cognitive function score and presence of cognitive decline at V2 and these effects were stronger than single‐time point estimates.

**Conclusion:**

Biological aging is associated with lower cognitive function score and greater cognitive decline in diverse Hispanic and Latino adults. Longitudinal assessment of change in age acceleration for second generations clocks, GrimAge and PhenoAge may provide additional value in predicting cognitive decline beyond single time point assessment.